# Vaccine candidates derived from a novel infectious cDNA clone of an American genotype dengue virus type 2

**DOI:** 10.1186/1471-2334-4-39

**Published:** 2004-10-04

**Authors:** Joseph E Blaney, Christopher T Hanson, Kathryn A Hanley, Brian R Murphy, Stephen S Whitehead

**Affiliations:** 1 Laboratory of Infectious Diseases, National Institute of Allergy and Infectious Diseases, National Institutes of Health, Bethesda, Maryland, 20892 USA

## Abstract

**Background:**

A dengue virus type 2 (DEN-2 Tonga/74) isolated from a 1974 epidemic was characterized by mild illness and belongs to the American genotype of DEN-2 viruses. To prepare a vaccine candidate, a previously described 30 nucleotide deletion (Δ30) in the 3' untranslated region of DEN-4 has been engineered into the DEN-2 isolate.

**Methods:**

A full-length cDNA clone was generated from the DEN-2 virus and used to produce recombinant DEN-2 (rDEN-2) and rDEN2Δ30. Viruses were evaluated for replication in SCID mice transplanted with human hepatoma cells (SCID-HuH-7 mice), in mosquitoes, and in rhesus monkeys. Neutralizing antibody induction and protective efficacy were also assessed in rhesus monkeys.

**Results:**

The rDEN2Δ30 virus was ten-fold reduced in replication in SCID-HuH-7 mice when compared to the parent virus. The rDEN-2 viruses were not infectious for *Aedes *mosquitoes, but both readily infected *Toxorynchites *mosquitoes. In rhesus monkeys, rDEN2Δ30 appeared to be slightly attenuated when compared to the parent virus as measured by duration and peak of viremia and neutralizing antibody induction. A derivative of rDEN2Δ30, designated rDEN2Δ30-4995, was generated by incorporation of a point mutation previously identified in the NS3 gene of DEN-4 and was found to be more attenuated than rDEN2Δ30 in SCID-HuH-7 mice.

**Conclusions:**

The rDEN2Δ30 and rDEN2Δ30-4995 viruses can be considered for evaluation in humans and for inclusion in a tetravalent dengue vaccine.

## Background

The increased prevalence of disease caused by the mosquito-borne dengue (DEN) viruses (four serotypes; DEN-1 – DEN-4) has intensified the effort to generate a vaccine that would both confer protection and be economically feasible for use in countries with limited resources for healthcare [1]. Dengue fever and dengue hemorrhagic fever and shock (DHF/DSS) are a severe disease burden for tropical and semitropical countries inhabited by more than 2.5 billion people [2]. Risk factors for the more severe disease, DHF/DSS, include the strain of virus, age and genetic background of the host, and secondary infection by a DEN serotype different from that which caused the primary infection [2]. Increased risk associated with secondary infection by a different DEN serotype is believed to be caused both by increased virus replication resulting from antibody-dependent enhancement and by augmented immune activation induced by the secondary infection [3,4]. Typically, regions with DHF/DSS have all four DEN serotypes circulating simultaneously, and an effective DEN vaccine must contain a tetravalent formulation that confers protection against each of the four DEN serotypes.

Immunity to the DEN viruses is primarily mediated by neutralizing antibodies directed against the envelope (E) glycoprotein, and most vaccine strategies aim to induce antibody against this major protective antigen. Live attenuated tetravalent vaccines appear to be the best vaccine candidates since they are economical to manufacture and they induce long-term immunity with the live attenuated yellow fever virus vaccine serving as a successful model flavivirus vaccine [5]. Several strategies to produce live attenuated tetravalent vaccines are being pursued including attenuation of viruses by conventional passage in tissue culture or introduction of defined attenuating mutations into recombinant DEN viruses [6-9]. In addition, chimeric dengue viruses are being evaluated that contain the E protein of a DEN virus on a background of either an attenuated DEN virus from a different serotype or a more distantly related, but attenuated, flavivirus [10-12].

We have previously described attenuated and immunogenic monovalent vaccine candidates for DEN-1, DEN-2, DEN-3, and DEN-4 that were generated by two distinct recombinant methodologies. Using the first methodology, nucleotides 10478–10507 were deleted from the 3' UTR (Δ30) of a wild type cDNA clone for DEN-4 to generate a vaccine candidate, rDEN4Δ30, which is safe, attenuated, and immunogenic in rhesus monkeys and humans [13]. Incorporation of the Δ30 mutation into an infectious cDNA clone of DEN-1 wild type virus at a site homologous to that in DEN-4 attenuated DEN-1 for rhesus monkeys and is currently being evaluated in humans [14]. The Δ30 mutation did not confer attenuation upon DEN-3 for reasons that have not been defined [15]. Thus, this approach has yielded live attenuated virus vaccine candidates for both DEN-1 and DEN-4. Using a second methodology, antigenic chimeric viruses have been generated by replacing the membrane protein (M) and E structural genes of rDEN4Δ30 with those from DEN-2 or DEN-3 [12,15]. These antigenic chimeric viruses were attenuated and immunogenic in rhesus monkeys and represent vaccine candidates for DEN-2 and DEN-3. We have also described a set of point mutations that can attenuate wild type rDEN-4 for SCID mice transplanted with human liver cells (SCID-HuH-7) or for rhesus monkeys [16,17]. Such mutations identified in rDEN-4 could be introduced into conserved sites of cDNA clones for other DEN serotypes to fine-tune the level of attenuation of vaccine candidates.

We have found it prudent to pursue several strategies to develop a live attenuated virus vaccine for each dengue serotype recognizing that it has been a challenge to achieve a satisfactory balance between attenuation and immunogenicity [15,18-20]. Thus, in addition to the antigenic chimeric DEN-2 vaccine candidate described above, a second approach was pursued in the present study that involved the construction of an infectious cDNA clone of a wild type DEN-2 virus isolated in Tonga [21], and the generation of DEN-2 vaccine candidates by the sequential introduction of defined attenuating mutations into the recombinant version of the DEN-2 Tonga/74 wild type virus. The rDEN2Δ30 vaccine candidate was evaluated for replication in SCID-HuH-7 mice, mosquitoes, and rhesus monkeys. In addition, an attenuating point mutation, previously described in DEN-4, was introduced into the rDEN2Δ30 virus, and this rDEN2Δ30 derivative was characterized in SCID-HuH-7 mice.

## Methods

### Cells and viruses

Vero cells (African green monkey kidney) were propagated in OptiPro SFM (Invitrogen, Grand Island, NY) supplemented with 4 mM L-glutamine (Invitrogen). HuH-7 cells (human hepatoma) were maintained in D-MEM/F-12 (Invitrogen) supplemented with 10% fetal bovine serum (FBS), 1 mM L-glutamine and 0.05 mg/ml gentamicin (Invitrogen). C6/36 cells (Aedes albopictus mosquito cells) were maintained at 32°C in Minimal Essential Medium (MEM) containing Earle's salts and 25 mM HEPES buffer (Invitrogen) and supplemented with 10% FBS, 2 mM L-glutamine, and 0.1 mM non-essential amino acids (Invitrogen).

A dengue virus type 2 isolate, Tonga/74, was provided by Dr. Duane Gubler (CDC, Fort Collins, CO). The virus was isolated during a 1974 dengue outbreak in the South Pacific island of Tonga [21]. The virus was isolated by inoculation of patient sera into *Aedes albopictus *mosquitoes, and subsequent passage in C6/36 cells before determination of genomic sequence.

### Sequence analysis

Viral RNA was isolated from DEN-2 Tonga/74 wild type virus using the QIAamp Viral RNA mini kit (Qiagen, Valencia, CA). Reverse transcription was performed using random hexamer primers and the SuperScript First-Strand Synthesis System for RT-PCR (Invitrogen). Overlapping PCR fragments of approximately 2000 base pairs were generated using DEN-2 specific primers and Advantage cDNA polymerase (ClonTech, Palo Alto, CA). Both strands of the resulting PCR fragments were sequenced directly on a 3100 Genetic Analyzer (Applied Biosystems, Foster City, CA) using DEN-2 specific primers in BigDye terminator cycle sequencing reactions (Applied Biosystems) and the results were assembled into a consensus sequence. To determine the nucleotide sequence of the genomic 5' and 3' regions, the 5' cap nucleoside of the viral genome was removed with tobacco acid pyrophosphatase (Epicentre Technologies, Madison, WI), followed by circularization of the genome using RNA ligase (Epicentre Technologies). An RT-PCR fragment spanning the ligation junction was generated and sequenced using DEN-2 primers. For the DEN-2 Tonga/74 consensus sequence, GenBank accession number AY744147 was assigned.

### Genetic construction of rDEN-2 Tonga/74 cDNA clone

**Figure 1:**
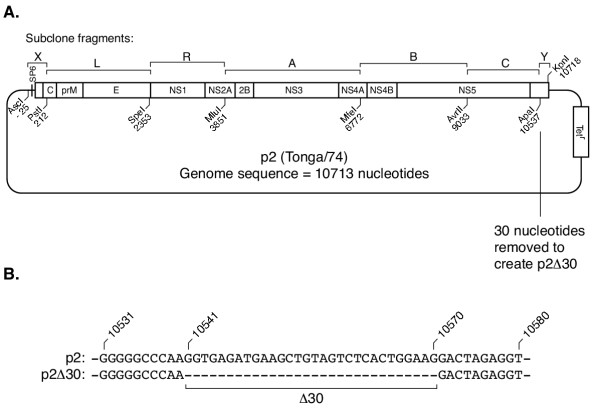
**Molecular construction of the DEN-2 full-length cDNA plasmids p2 and p2Δ30. A. **Diagram of the complete full-length DEN-2 Tonga/74 cDNA plasmid p2 is shown annotated with the restriction enzyme and corresponding cleavage site locations used to assemble the subcloned RT-PCR fragments. Restriction enzyme cleavage sites are numbered relative to nucleotide position in the virus genome. The corresponding genomic regions encoded by each subcloned RT-PCR fragment, X, L, R, A, B, C, and Y, are shown above the plasmid diagram. Relative positions of the SP6 promoter and tetracycline resistance gene (Tet^r^) are indicated. **B. **To generate plasmid p2Δ30, 30 nucleotides are removed from the 3'-UTR (Fragment Y). The nucleotide sequence encompassing the Δ30 region is shown for the p2 parent cDNA and the resulting p2Δ30 cDNA. Nucleotide positions in the virus genome are indicated for p2.

cDNA fragments of DEN-2 Tonga/74 were generated by reverse-transcription of the genome as indicated in Figure 1. Each fragment was subcloned into a plasmid vector and sequenced to verify that it matched the consensus sequence as determined for the virus. This yielded seven cloned cDNA fragments spanning the genome. Cloned fragments were modified as follows: Fragment X, representing the 5' end of the genome was abutted to the SP6 promoter; Fragment L was modified to contain a *Spe*I restriction site at genomic nucleotide 2353; Fragment R was modified to contain a *Spe*I restriction site also at genomic nucleotide 2353, and, to stabilize the eventual full-length clone, two additional mutations at nucleotides 2362 – 2364 and 2397 were created to ensure that translation stop codons were present in all reading frames other than that used to synthesize the virus polyprotein; Fragment A was modified at nucleotide 3582 to ablate a naturally occurring *Spe*I restriction site and at nucleotide 4497 to ablate a naturally occurring *Kpn*I restriction site; Fragment C was modified at nucleotide 9374 to ablate a naturally occurring *Kpn*I restriction site; and Fragment Y, representing the 3' end of the genome was abutted to a *Kpn*I restriction site. All mutations introduced into the cloned cDNA fragments were translationally-silent, thereby preserving the wild-type polyprotein sequence. Each fragment was added incrementally between the *Asc*I and *Kpn*I restriction sites of DEN-4 cDNA clone p4 (GenBank accession number: AY648301) to generate a full-length DEN-2 cDNA clone (p2) with the same vector background successfully used to generate rDEN-4 and rDEN4Δ30 virus [13]. cDNA clone p2 was sequenced to confirm that the virus genome region matched the DEN-2 Tonga/74 consensus amino acid and nucleotide sequence, with the exception of the translationally-silent modifications noted above. The Δ30 mutation which removes nucleotides 10541–10570 was introduced into Fragment Y to generate Fragment YΔ30. To create p2Δ30, the Fragment Y region of p2 was replaced with Fragment YΔ30 (Figure 1). The genomic region of each full-length cDNA was sequenced as described above and GenBank accessions were assigned as follows (cDNA clone: accession numbers): p2: AY744148, p2Δ30: AY744149.

Using site-directed mutagenesis, an attenuating amino acid change characterized in the NS3 gene of DEN-4 (nt 4995–7; a.a. 158, Ser→Leu) was introduced into the p2Δ30 cDNA clone [17]. A mutagenic oligonucleotide was designed to change DEN-2 NS3 amino acid 158 from Ser (AGT) to Leu (CTA) and used to construct the cDNA clone, p2Δ30-4995 (accession number: AY744150), which was sequenced for confirmation of nucleotide changes.

### Recovery of rDEN-2 viruses

cDNA clones were linearized with *Acc*65I (isoschizomer of *Kpn*I which cleaves leaving only a single 3' nucleotide) and were transcribed *in vitro *using the AmpliCap SP6 Message Maker kit (Epicentre Technologies, Madison, WI). Purified transcripts were then transfected into Vero or C6/36 cells. Viruses recovered in C6/36 cells were passaged 3 times in Vero cells, and all viruses were biologically cloned by terminal dilution in Vero cells. The genomes of recombinant viruses used to infect rhesus monkeys were completely sequenced as described above to identify adventitious mutations that had accumulated during transfection and biological cloning.

### Replication in SCID-HuH-7 mice

Four to six week-old SCID mice (Tac:Icr:Ha(ICR)-*Prkdc*^*scid*^) (Taconic, Germantown, NY) were injected intraperitoneally with 10^7 ^HuH-7 cells suspended in 0.2 ml phosphate-buffered saline. Tumors were detected in the peritoneum, and mice were infected by direct inoculation of the tumor with 10^4 ^PFU of virus in 0.05 ml Opti-MEM (Invitrogen). On day 7 post-infection, serum was obtained from cardiac blood and stored at -70°C. Virus titer in serum samples was determined by plaque assay in Vero cells.

### Replication, immunogenicity, and protection in rhesus monkeys

The DEN-2 viruses were evaluated in rhesus macaques using established methods [13]. DEN virus sero-negative monkeys were injected subcutaneously with 10^5 ^PFU virus diluted in L-15 medium (Invitrogen) or with a mock inoculum. Serum was collected on days 0–6, 8, 10, 12 and 28 after inoculation and stored at -70°C. Virus titer was determined for each serum sample by plaque assay in Vero cells and serum neutralizing antibody titer was determined for serum from days 0 and 28 by plaque reduction neutralization test. On day 28, monkeys were challenged with 10^5 ^PFU of DEN-2 Tonga/74, and serum was collected on days 29–34, 36, and 56. Virus titer was determined for serum from days 28–34 and 36 and serum neutralizing antibody titer was determined for serum from day 56.

### Virus replication in mosquitoes

Replication in *Aedes aegypti *and *Toxorynchites amboinensis *mosquitoes was evaluated as previously described [22]. Briefly, *A. aegypti *were fed blood meals containing serial 10-fold dilutions of virus. After 21 days, viral antigen was detected in head and midgut preparations by immunoflourescence assay using DEN-2-specific hyperimmune mouse ascitic fluid and fluorescein isothyocyanate conjugated goat anti-mouse IgG (KPL, Gaithersburg, MD), and the mosquito infectious dose-50% (MID_50_) was determined. *T. amboinensis *were inoculated intrathoracically with a 0.2 ul dose containing serial ten-fold dilutions of virus and incubated for 14 days. Head preparations were made and antigen visualized as described above.

## Results

### Generation and sequence analysis of recombinant DEN-2 Tonga/74 viruses

A full-length cDNA clone, p2, was constructed that matched the genomic consensus sequence of the American genotype DEN-2 isolate, Tonga/74, with the exception of translationally-silent modifications made to facilitate cloning (Figure 1). The previously described Δ30 deletion mutation was incorporated into the p2 cDNA clone to form p2Δ30 [13]. The rDEN-2 virus was recovered in C6/36 and Vero cells, but the presence of the Δ30 mutation limited recovery to only C6/36 cells. After passage in Vero cells, adaptation mutations were identified by sequence analysis as had been described for other DEN viruses [23]. Both rDEN-2 and rDEN2Δ30 viruses accumulated a single nucleotide change in NS4B at nt 7169 encoding a Val→Ala change at amino acid position 115 as has been observed for rDEN-3 (Table 1) [15]. The same nucleotide change was previously reported to occur at the homologous site following passage of DEN-4 in Vero cells resulting in a Leu→Ser change (Table 1) [23]. Inclusion of the 7169 mutation into the p2Δ30 cDNA permitted recovery in both C6/36 and Vero cells (data not shown). The rDEN2Δ30 virus reached a virus titer of 6.6 log_10_PFU/ml in Vero cells.

### Replication of rDEN-2 viruses in SCID-HuH-7 mice

As an initial evaluation of replication of the DEN-2 Tonga/74 virus and the rDEN-2 viruses, replication in SCID mice transplanted with HuH-7 human hepatoma cells (SCID-HuH-7 mice) was tested. Wild-type viruses from each DEN serotype have been shown to replicate to approximately 6.0 log_10_PFU/ml serum in SCID-HuH-7 mice, and an *att *phenotype in SCID-HuH-7 mice has been shown to be a predictor of reduced replication in rhesus monkeys [12,14,15,17]. The parent DEN-2 Tonga/74 virus replicated efficiently in SCID-HuH-7 mice and reached a mean titer in serum of 5.9 log_10_PFU/ml (Table 2) similar to that previously observed with the DEN-2 New Guinea C (NGC) prototype strain [12]. The rDEN-2 virus replicated to the same level as the wild-type isolate, while rDEN2Δ30 was 10-fold restricted in replication. This reduction was statistically significant (Tukey-Kramer post-hoc test; *P *< 0.05), and was similar to that observed for the well-characterized rDEN4Δ30 virus [17].

### Replication of rDEN-2 viruses in mosquitoes

The DEN-2 viruses were evaluated for infectivity of *Aedes aegypti *fed on an infectious bloodmeal (oral infectivity only) and for *Toxorynchites amboinensis *inoculated intrathoracically (Table 3). At the doses tested neither DEN-2, rDEN-2, or rDEN2Δ30 were detected in the midgut or head of *A. aegypti *mosquitoes which had fed on an infectious bloodmeal 21 days earlier. The inability to infect the midgut led to a lack of infection in the head tissue. This indicates that the DEN-2 Tonga/74 viruses are poorly infectious for *A. aegypti *mosquitoes by oral infectivity, as has been demonstrated for multiple DEN-2 viruses of the American genotype [24,25]. In contrast the DEN-2 NGC prototype strain, an Asian genotype member, was highly infectious in *A. aegypti *mosquitoes when tested previously but it was not included here as a concurrent control [12].

The defect in rDEN-2 infectivity for *A. aegypti *was further investigated by directly inoculating the same virus stocks intrathoracically into *T. amboinensis *and measuring the ability of the viruses to infect the head tissues. Both rDEN-2 and rDEN2Δ30 were highly infectious by intrathoracic inoculation (Table 3). The Δ30 mutation did not alter the infectivity of rDEN-2 following intrathoracic inoculation, a property also previously observed for DEN-1, -3 and -4 [14,15,22]. These results indicate that the lack of infectivity for *A. aegypti *was likely caused by the inability of the DEN-2 Tonga/74 viruses to establish a midgut infection and that the viruses retained the ability to infect head tissues.

### Replication, immunogenicity, and protective efficacy in rhesus monkeys

The replication (viremia), immunogenicity, and protective efficacy of the DEN-2 viruses in monkeys were studied. Monkeys inoculated with the DEN-2 Tonga/74 wild-type isolate were viremic for an average of 4.5 days with a mean peak titer of 2.1 log_10_PFU/ml (Table 4). Inoculation with rDEN-2 resulted in detectable viremia for 4.0 days with a mean peak titer of 1.9 log_10_PFU/ml. While the levels of rDEN2Δ30 replication (2.8 days viremia; mean peak titer of 1.7 log_10_PFU/ml) were lower than DEN-2 and rDEN-2, the differences were not as dramatic as had been observed for rDEN1Δ30 and rDEN4Δ30 when compared to their parent viruses [13,14]. The level of neutralizing antibodies induced by the rDEN2Δ30 virus was also less than that induced by the wild-type DEN-2 viruses, a finding consistent with the decreased replication exhibited by this vaccine candidate. Therefore, by three quantitative measures, duration and peak titer of viremia and the level of neutralizing antibodies induced, rDEN2Δ30 appeared to be attenuated when compared to DEN-2 Tonga/74. When vaccinated monkeys were challenged with DEN-2 Tonga/74, all monkeys were protected, as indicated by the lack of viremia (Table 4).

### The 4995 mutation further attenuates rDEN2Δ30 in SCID-HuH-7 mice

Based on the limited attenuation conferred upon rDEN-2 by the Δ30 mutation in rhesus monkeys, we sought to construct a further attenuated derivative of rDEN2Δ30. To further attenuate rDEN2Δ30, an *att *mutation that has been characterized in another DEN serotype was imported into a homologous region in DEN-2. One such mutation, the 4995 mutation in DEN-4 NS3 at amino acid 158 (Ser→Leu), was previously incorporated into the DEN-4 vaccine candidate, rDEN4Δ30, and found to further attenuate the virus for SCID-HuH-7 mice and rhesus monkeys [17]. Site directed mutagenesis was used to introduce a Ser→Leu mutation at amino acid 158 of NS3 in rDEN2Δ30-7169, and the rDEN2Δ30-4995 virus was recovered in C6/36 cells and propagated in Vero cells reaching a virus titer of 6.2 log_10_PFU/ml (Table 1). Importantly, the resulting Leu codon would require two nucleotide changes to revert to one of the six odons encoding a Ser residue.

Replication in the SCID-HuH-7 mouse model was used as an initial assessment of the rDEN2Δ30-4995 virus phenotype. Table 5 includes results from three separate experiments (including those from Table 1) and confirms the approximate 10-fold reduction in replication conferred by the Δ30 mutation upon rDEN-2 replication in SCID-HuH-7 mice. The rDEN2Δ30-4995 virus had a mean peak virus titer of 4.6 log_10_PFU/ml which was only a modest reduction from that of rDEN2Δ30, 5.2 log_10_PFU/ml. However, comparison of a large number of samples indicated that the reduction in virus titer conferred by the NS3 4995 mutation upon rDEN2Δ30 was statistically significant (rDEN2Δ30-4995 versus rDEN2Δ30; Tukey-Kramer post-hoc test; *P *< 0.05). The virus titer of rDEN2Δ30-4995 virus in SCID-HuH-7 mice was over 60-fold reduced from that of the rDEN-2 parent virus.

## Discussion

Development of a live-attenuated tetravalent dengue vaccine has been complicated by two major factors. First, monovalent vaccine candidates that exhibit a satisfactory balance between attenuation and immunogenicity have been difficult to identify [15,18-20]. Second, satisfactorily attenuated tetravalent vaccine formulations that induce a broad neutralizing antibody response against each of the four DEN serotypes have been difficult to develop [6,10,20,26]. For these reasons, we have sought to develop multiple vaccine candidates for each DEN serotype to increase the likelihood that a vaccine with a satisfactory balance between attenuation and immunogenicity will be identified. To produce a live-attenuated DEN-2 vaccine candidate, we previously generated an antigenic chimeric virus, rDEN2/4Δ30, expressing the M and E structural genes of the DEN-2 NGC strain on the attenuated rDEN4Δ30 background [12]. The vaccine candidates described in the present study, rDEN2Δ30 and rDEN2Δ30-4995, could serve as alternates to this antigenic chimeric virus if evaluation of the rDEN2/4Δ30 virus in humans, either as a monovalent vaccine or as a component of a tetravalent vaccine, indicates that it lacks a balance between attenuation and immunogenicity.

It was hoped that each of the four components of a tetravalent vaccine, consisting of DEN-1, -2, -3, and -4 wild type viruses, each with the common 30 nucleotide deletion mutation in the 3' UTR, would exhibit a similar level of attenuation in animal models [13-15]. Unfortunately, the level of attenuation conferred by the Δ30 mutation upon each of the four serotypes has proven to be variable. In rhesus monkeys, the rDEN2Δ30 virus appears to have an intermediate attenuation phenotype in between that of the attenuated rDEN1Δ30 and rDEN4Δ30 and the non-attenuated rDEN3Δ30 [13-15]. Although rDEN2Δ30 was slightly attenuated compared to its DEN-2 parent virus in rhesus monkeys, the reduction in replication was less than that of rDEN1Δ30 and rDEN4Δ30. While the latter two viruses had detectable viremia in only 50% of monkeys, a mean number of viremic days of less than one day, and a mean peak viremia of less than 1.0 log_10_PFU/ml [13,14], the rDEN2Δ30 virus infected 100% of the rhesus monkeys and reached a peak virus titer of 1.7 log_10_PFU/ml. However, the 10-fold reduction of replication of rDEN4Δ30 and rDEN2Δ30 in SCID-HuH-7 mice, compared to that of their respective wild type parents, was similar. To date, rDEN4Δ30 is the only Δ30 vaccine candidate that has been tested in humans, and it was found to be both safe and immunogenic [13]. If the level of attenuation in SCID-HuH-7 mice serves as a better guide to attenuation in humans, rDEN2Δ30 might be satisfactorily attenuated in humans since its level of attenuation for SCID-HuH-7 mice and that of the rDEN4Δ30 vaccine candidate are comparable.

To construct a further attenuated derivative of rDEN2Δ30, the 4995 mutation present in the NS3 gene of DEN-4 at amino acid 158 (Ser→Leu) was introduced into the homologous region of the NS3 protein of rDEN2Δ30 [17]. Although the 4995 mutation results in a single amino acid change and thus may be susceptible to reversion, the mutant leucine codon selected for insertion into rDEN2Δ30-4995 would require two nucleotide changes to revert to a serine codon. Introduction of the 4995 mutation into rDEN4Δ30 resulted in a 100-fold greater reduction of replication in SCID-HuH-7 mice [17]. In rDEN2Δ30, its introduction resulted in nearly a 10-fold reduction in virus titer, a smaller but still statistically significant reduction. These results provide a second example of the difficulty in predicting the precise level of attenuation following import of an attenuating mutation into a different DEN serotype. Nevertheless, the rDEN2Δ30-4995 vaccine candidate is more attenuated than its rDEN2Δ30 parent and warrants evaluation in rhesus monkeys and humans.

Epidemiologic and molecular pathogenesis studies of DEN-2 strains support the concept that the DEN-2 Tonga/74 virus, from which the vaccine candidates were derived, may naturally have a lower level of virulence than other DEN-2 viruses. If the DEN-2 Tonga/74 parent virus is naturally attenuated to some degree, only a small incremental increase in attenuation might be required to satisfactorily attenuate it for humans. Gubler et al. investigated the 1974 outbreak of DEN-2 infection in the Pacific island of Tonga [21]. In comparison to a subsequent DEN-1 outbreak, the 1974 DEN-2 outbreak was distinguished by mild disease with few hemorrhagic sequelae, low viremia, and an overall slow spread of virus infection [21]. The weak DEN-2 outbreak was proposed to be a result of the circulation of a strain with an inherently low level of virulence [21].

Since the Tonga/74 outbreak, additional evidence has emerged that supports the suggestion that there are at least two circulating lineages of DEN-2 viruses that differ in virulence [27-29]. The DEN-2 Tonga/74 virus is a member of the DEN-2 American genotype, which as a group appear to possess lower virulence than that of the Asian genotype of DEN-2 viruses [28,29]. Despite the presence of the American DEN-2 genotype viruses and limited co-circulation of DEN-1 and DEN-3 viruses in the Americas in the 1960s and 1970s, the first major epidemic of DHF/DSS in the Americas occurred only after the introduction of a DEN-2 Asian genotype virus in 1981 [27-30]. It was thought that genetic differences might have contributed to this difference in virulence and evidence to this effect has been forthcoming. Rico-Hesse and colleagues have defined genetic elements within the genome of DEN-2 American genotype viruses which distinguish them from members of the Asian genotype [31]. In addition, using chimeric rDEN-2 American/Asian viruses, introduction of three genetic elements (a point mutation in the E gene, the 5' UTR, and the 3' UTR) of the American genotype was found to confer reduced virus replication in dendritic cells and monocytes upon an Asian genotype rDEN-2 [32]. The Tonga/74 virus shares each of these three attenuating genetic determinants specific to the American genotype [31,32], which provides a possible explanation for its lower virulence in humans. The rDEN2Δ30 vaccine candidate, whose parent is the DEN-2 Tonga/74 American genotype virus, thus contains naturally occurring and experimentally introduced attenuating mutations. Thus, the small incremental increase in attenuation provided by the Δ30, with or without the 4995 mutation, might prove to satisfactorily attenuate the DEN-2 Tonga/74 for humans.

The American genotype DEN-2 viruses exhibit decreased infectivity for *Aedes *mosquitoes in comparison to Asian DEN-2 viruses [24,25]. Consistent with these observations, the wild type New Guinea C Asian DEN2 virus was highly infectious for *Aedes *mosquitoes in our laboratory [12] whereas the Tonga/74 American genotype virus was poorly infectious by the oral route (present study). In fact, the increased prevalence of DEN-2 viruses of the Asian genotype in the Americas has been suggested to be a result of their enhanced transmission [28]. However, the active circulation of American genotype viruses over many decades indicates that mosquito transmission does occur and large epidemics have been associated with viruses of this genotype [21,27]. At the doses tested, neither the DEN-2 Tonga/74 isolate nor the recombinant viruses were found to infect the midgut or head of *Aedes aegypti *mosquitoes fed an infectious blood meal. Since rDEN-2 and rDEN2Δ30 viruses were infectious by intrathoracic inoculation of *Toxorynchites *mosquitoes, the lack of infectivity for *A. aegypti *was likely caused solely by the inability of the DEN-2 Tonga/74 viruses to establish a midgut infection. Decreased infectivity for *Aedes *mosquitoes could serve to help limit transmission of the vaccine virus.

## Conclusions

The live-attenuated DEN-2 virus candidates described here, rDEN2Δ30 and rDEN2Δ30-4995, have several properties desired in a live attenuated virus vaccine for humans. First, both viruses reached a titer over 6.0 log_10_PFU/ml in Vero cells that would permit economical manufacture. Second, the viruses are derived from the DEN-2 Tonga/74 strain, a member of the American genotype, which has been associated with decreased virulence. Third, rDEN2Δ30 was attenuated for replication in SCID-HuH-7 mice and slightly attenuated for rhesus monkeys while inducing a protective neutralizing antibody response. Fourth, rDEN2Δ30-4995 was more attenuated in SCID-HuH-7 mice than rDEN2Δ30. Fifth, the DEN-2 Tonga/74 strain, like other members of the American genotype, is poorly infectious for *Aedes aegypti *mosquitoes which would help to limit uncontrolled transmission of the vaccine virus.

## Competing interests

The authors declare that they have no competing interests.

## Authors' contributions

J.B. recovered viruses, conducted animal studies, and drafted the manuscript. C.H. and S.W. constructed the DEN-2 cDNA clone and C.H. performed sequencing. K.H. performed mosquito studies. B.M. and S.W. supervised the study and participated in planning and design. All authors read and approved the manuscript.

## Pre-publication history

The pre-publication history for this paper can be accessed here:



## Figures and Tables

**Table 1:** Missense and UTR mutations which arose spontaneously in rDEN-2 and rDEN2Δ30 viruses during passage in Vero cells.

Virus	Gene	Nucleotide position	Nucleotide substitution	Amino acid position^a^	Amino acid change
rDEN-2	NS4B	7169^b^	U > C	115	Val > Ala
	NS5	9248	A > C	560	Glu > Ala
rDEN2Δ30	NS4B	7169^b^	U > C	115	Val > Ala
rDEN2Δ30-4995	NS3	4949	A > G	143	Lys > Arg
	NS4B	7169^b^	U > C	115	Val > Ala
	3' UTR	10322	G > A	--	--

^a ^Amino acid position in the individual DEN-2 protein.

^b ^Nucleotide position 7169 in DEN-2 corresponds to nt 7162 in rDEN-4, and the wild-type amino acid is Val and Leu, respectively [23].

**Table 2:** Addition of the Δ30 mutation to rDEN-2 decreases replication in SCID-HuH-7 mice by 10-fold.

	Replication in SCID-HuH-7 mice^a^	
	
Virus	No. of mice	Mean virus titer (log_10_PFU/ml ± SE)
DEN-2 Tonga/74	6	5.9 ± 0.3
rDEN-2	7	5.9 ± 0.2
rDEN2Δ30	9	4.9 ± 0.2

^a ^Groups of HuH-7-SCID mice were inoculated directly into the tumor with 10^4 ^PFU virus. Serum was collected on day 7 and tittered in Vero cells.

**Table 3:** DEN-2 Tonga/74 is poorly infectious for *Aedes aegypti *fed an infectious bloodmeal, and the Δ30 mutation does not decrease infectivity for *Toxorynchites amboinensis*.

	*A. aegypti *(oral infection)				*T. amboinensis *(intrathoracic inoculation)		
		
Virus	Dose (log_10_PFU)^a^	No. tested	% Infected^b^		Dose (log_10_PFU)^c^	No. tested	% Infected^d^
					
			Midgut	Head			
DEN-2 Tonga/74	2.8	20	0	0	--	--	--
rDEN-2	3.5	18	0	0	2.9	5	100
					1.9	12	83
					0.9	8	63
					-0.1	7	14
					-1.1	7	0
							MID_50 _= 0.9
rDEN2Δ30	3.5	22	0	0	2.7	8	100
					1.7	7	100
					0.7	6	83
					-0.3	7	43
					-1.3	4	50
					-2.3	5	0
							MID_50 _= -0.2

^a ^Amount of virus ingested, assuming a 2 ul bloodmeal.

^b ^Percentage of mosquitoes with antigen detectable by IFA in midgut or head tissue prepared 21 days after oral infection.

^c ^Amount of virus present in 0.22 ul inoculum.

^d ^Percentage of mosquitoes with antigen detectable by IFA in head tissue prepared 14 days post-inoculation.

**Table 4:** Replication and immunogenicity of DEN-2 viruses in rhesus monkeys

Virus^a^	% with viremia	Mean no. of viremic days per monkey^b^	Mean peak virus titer (log_10_PFU/ml ± SE)	Geometric mean serum neutralizing antibody titer on day 28 (reciprocal dilution)^c^	Virus replication after challenge^d^	
					
					% with viremia	Mean peak virus titer (log_10_PFU/ml ± SE)
Mock	0	0	<0.7	<10	100	2.1 ± 0.1
DEN-2 Tonga/74	100	4.5	2.1 ± 0.3	311	0	<0.7
rDEN-2	100	4.0	1.9 ± 0.1	173	0	<0.7
rDEN2Δ30	100	2.8	1.7 ± 0.2	91	0	<0.7

^a ^Groups of rhesus monkeys (mock: n = 2; viruses: n = 4) were inoculated subcutaneously with 10^5 ^PFU of the indicated virus in a 1 ml dose. Serum was collected daily for day 0 to 6; day 8, 10, 12 and 28

^b ^Virus titer in serum was determined by plaque assay in Vero cells. The lower limit of detection was 0.7 log_10_PFU/ml. Viremia was not detected in any monkey after day 6.

^c ^Plaque reduction (60%) neutralizing antibody titers were determined using DEN-2 Tonga/74. Limit of detection was < 1:10. All monkeys inoculated with virus had a four-fold or greater increase in neutralizing antibody titer.

^d ^Vaccinated rhesus monkeys were inoculated subcutaneously with 10^5 ^PFU of DEN-2 Tonga/74 virus in a 1 ml dose. Serum was collected daily for day 0 to 8.

**Table 5:** Addition of the 4995 mutation further attenuates rDEN2Δ30 in SCID-HuH-7 mice.

	Replication in SCID-HuH-7 mice^a^	
	
Virus	No. of mice	Mean virus titer (log_10_PFU/ml ± SE)
DEN-2 Tonga/74	6	5.9 ± 0.3
rDEN-2	15	6.4 ± 0.2
rDEN2Δ30	22	5.2 ± 0.1
rDEN2Δ30-4995	28	4.6 ± 0.2

^a ^Groups of HuH-7-SCID mice were inoculated directly into the tumor with 10^4 ^PFU virus. Serum was collected on day 7 and titered in Vero cells. Data is included from three separate experiments.
